# Health related quality of life among pregnant women living with HIV in Kenya, results from comparing a patient generated index and the Euroqol 5 dimension 3 level

**DOI:** 10.1186/s12905-022-01646-9

**Published:** 2022-03-09

**Authors:** Jonathan Mwangi, Laura Ternent, Patricia Opondo Awiti Ujiji, Edwin Were, Anna Mia Ekström

**Affiliations:** 1 Department of Global Public Health, Karolinska Institutet, Stockholm, Sweden; 2 Population Health Sciences Institute, Newcastle University, Newcastle upon Tyne, UK; 3 Moi University, Eldoret, Kenya

## Abstract

**Background:**

Standardized tools are used to measure health-related quality of life (HRQoL) and they focus on selected physical, emotional, and social functioning. This approach may miss out on the heterogeneity of HRQoL among various sub-populations. The patient-generated index (PGI) is a tool used to measure HRQoL based on patients' expectations. Among patients living with HIV, HRQoL is an important indicator as the world moves beyond the UNAIDS 90-90-90 goals, towards the so-called fourth 90 that aims at good HRQoL. We compared the PGI and the Euroqol 5 Dimension 3-level (EQ-5D-3L) to identify areas of importance to pregnant women living with HIV affecting thier HRQoL.

**Methods:**

Through convenience sampling, we surveyed 100 pregnant women living with HIV attending antenatal and postnatal clinics in Western Kenya, using both the PGI and the EQ-5D-3L questionnaires. A PGI score and EQ-5D-3L index were generated for each participant. Data from the PGI was also summarized into themes. The PGI scores and EQ-5D-3L index scores were correlated using Pearson correlation.

**Results:**

From the PGI tool, 64% of the women reported having two to three main priority areas of their lives affected by their HIV status. These areas centered on themes of economic wellbeing (84% of the women), physical health (58%), psychological/emotional health (49%), and relationships (28%). The mean PGI score was 2.01 [SD = 1.10; median 1.10]. The majority of the women reported having no problems in any of the 5 dimensions captured in the EQ-5D-3L. The mean EQ-5D-3L score was 0.94 [SD = 1.10; median 1.00]. Both the EQ-5D-3L and the PGI showed less than perfect HRQoL. There was no correlation between the PGI and the EQ-5D-3L scores.

**Conclusion:**

The PGI may capture aspects of contextual social and emotional life for Kenyan pregnant women living with HIV that are not identified by generic tools. Highlighting areas of importance to patients’ HRQoL is key as focus shifts towards the fourth 90 and may also inform the design of care programs aligned to patient needs.

## Background

Assessing health-related quality of life (HRQoL) is critical in evaluating the impact of diseases on individuals’ lives [1]. HRQoL is described as a focus on the aspects of quality of life affected by health states [2]. However, controversy remains on the definition of HRQoL [3]. Karimi et al. (2016) argue that some definitions fail to distinguish between HRQoL and health or between HRQoL and quality of life (QoL), particularly because HRQoL questionnaires measure self-perceived health status [4]. The World Health Organization (WHO) defines QoL as “an individual's perception of their position in life in the context of the culture and value systems in which they live and in relation to their goals, expectations, standards, and concerns.” Among patients living with HIV, assessing HRQoL is gaining prominence as we look beyond the UNAIDS’ 90-90-90 goal of knowing one's status, being on treatment, and achieving viral suppression, to include a fourth 90 that considers HRQoL of people living with HIV [5, 6]. Among pregnant women living with HIV, overall maternal wellbeing may impact adherence to antiretroviral therapy and this may increase the risk of transmission of HIV from the mother to the new born [7].

Most of the generic methods used to measure HRQoL utilize tools that focus on selected physical, emotional and social functioning [8, 9]. These tools are developed from consensus or panels of experts from the early Karnofsky performance scale [10] and are often validated in high-income countries. This presents challenges when measuring HRQoL among populations in low- and middle-income countries [11], especially in settings where they have not been validated. These tools may also be broadly categorized as either disease-specific tools that are tailored to monitor specific outcomes that affect a specific disease process, or generic tools that measure generic outcomes across different diseases [12]. Disease-specific tools have the advantage of focusing on specific aspects of QoL affected by the disease and also on those aspects monitored by clinicians [12], but may not be suitable for comparing HRQoL across different health conditions or in patients with multiple illnesses [9]. Non-specific (generic) HRQoL tools, on the other hand, enable comparison across different diseases and an opportunity to compare the impact of different health interventions [12], including utility measures in economic evaluations [13]. This serves multiple purposes such as public health decision-making and resource allocation. One such generic tool is the EuroQol 5 dimension 3 level (EQ-5D 3L), a widely used tool developed by the EuroQol group to concisely measure and compare health status across different diseases [14]. However, the EQ-5D 3L and other standardized or disease-specific tools have been shown to not fully represent patients’ health status [12, 15]. In particular, they may miss out on subjective patient experiences of living with a certain health condition [16]. This thus underlines the need for use of different tools that complement each other in describing patients’ HRQoL [9, 17].

The patient-generated index (PGI) is a tool used to measure HRQoL, based on patients' expectations [18]. It is drawn from a theoretical model and provides a conceptual framework for the definition of HRQoL, that allows patients to formulate their measures [18]. The PGI has been used in diverse groups of patients including patients with cancer [19, 20], rheumatoid arthritis [21], systemic sclerosis [22] as well as people living with HIV in Thailand [23]. It is described as being more responsive to the patient’s perceptions of HRQoL compared with the standardized tools [24]. With the heavy burden of HIV in Sub-Saharan Africa, the use of the PGI to describe HRQoL among people living with HIV in Africa could potentially reveal key insights into what really matters from their perspective. Importantly among pregnant women living with HIV, efforts to eliminate perinatal transmission of HIV warrant a holistic approach in the assessment of their wellbeing. Our study aims to compare the utility of the EQ-5D-3L and the PGI among pregnant women living with HIV in Kenya, in a bid to provide a better understanding of HRQoL in this population..

## Methods

The study was nested within a randomized intervention, the “WelTel PMTCT”, a trial investigating the effect of weekly text messaging in improving 2-year retention in care across prevention of mother to child transmission of HIV (PMTCT) programs [25].

### Study setting and population

The study was carried out in Western Kenya in four public health facilities. Western Kenya bears a great part of the HIV burden in Kenya with an HIV prevalence as high as 20% in some counties [26]. However, great improvement in ART coverage has been noted since access to antiretroviral therapy (ART) was scaled up over the past 5 years [27]. Public primary healthcare clinics in Kenya routinely offer PMTCT services, integrated with other antenatal and postnatal services.

### Eligibility criteria

Pregnant women (irrespective of gestational age) aged above 18 years, living with HIV, regardless of time since diagnosis, and regardless of whether they had been initiated on ART or not, who gave written informed consent to participate, were eligible for the study.

### Study participants

From the 600 participants enrolled in the Weltel PMTCT trial [24] convenience sampling was used to enrol 100 participants. The sample size of 100 participants was based on other published studies with the PGI [28, 29]. Pregnant women living with HIV who fulfilled the eligibility criteria were consecutively recruited and participated in a structured interview. Mentor mothers and nurses providing routine care at the clinics enrolling patients in the WelTel trial, described the study to all women attending the clinic, most frequently those who were waiting in line to be attended to by health personnel for routine checkup or ART refill. A participant who had agreed to take part in the PGI study interview was escorted to a private space within the same clinic for the interview by the research assistants (RAs) who had obtained informed consent from the woman. This was to offer privacy during the interview as well as minimize service delivery interruptions at the clinic.

### Data collection

Between July 2015 to April 2016, 100 study participants were interviewed using both the using the PGI and EQ-5D-3L. They participated in a structured interview where the PGI and EQ-5D-3L questionnaires were administered by trained RAs familiar with the local healthcare system and socio-cultural environment. The principal investigator (JM) trained the RAs on both the PGI and the EQ-5D-3L tools before the study. Piloting of the study tools had also been done by the prinicipal investigator before the trial among 10 pregnant women living with HIV attending a clinic at Kibera, an urban informal settlement with high HIV prevalence in Nairobi, Kenya.

The RAs administered the questionnaires mainly in Kiswahili and English and then filled out the responses of the study participants into the questionnaires. The PGI tool was completed in three stages through the interviewer-administered questionnaire. In the first stage each participant was asked to list the five most important areas or activities in their lives affected by their HIV infection. In the second stage, each participant was asked to rate how badly affected their lives were in each of their chosen areas, on a numeric scale of 0–6, with zero (0) being the worst they can imagine, to six (6) being as good as they could be. In the third stage, each participant was asked to imagine that they had an opportunity to improve affected areas of their lives. They were given a total of imaginary 10 points to “spend” on improving the areas they had identified as affected by their condition in stage one. The total points allocated across all areas could not exceed ten points. If no points were allocated, it meant a patient would like the area to remain exactly as it was. The points they allocated indicated the relative importance of potential improvements in that area.

After the PGI interview, the EQ-5D-3L was also administered to the study participants by the interviewer, during the session.. The EQ-5D-3L questionnaire consists of five dimensions: mobility, self-care, usual activities, pain/discomfort, and anxiety/depression, each dimension with three levels: no problems, some problems, and extreme problems. For each of these dimensions the participants indicated their health corresponding to one of the 3 levels, indicated by a digit. For the 5 dimensions, the resulting combined 5 digit number corresponded to the patients’ health state which was converted to an index score using validated weights. The resulting EQ-5D-3L index score ranged from 0–1; 0 being worst imaginable health and 1 being full health..

### Data analysis

Data was analyzed using R^©^ (2016 version 3.2.5).

The socio-demographic characteristics of the study participants were described using means, proportions, and percentages.

For the PGI, the index score for each participant was generated by taking each area of importance listed in stage 1, multiplying the ratings assigned in stage 2 by the proportion of points allocated in stage 3, generating an index score between 0–6. An illustration of the scoring is provided in Table 1.

**Table 1:** PGI score computation for a study participant

Stage 1: Area or activity affected	Stage 2: Score out of 6		Stage 3: Spend 10 points	Score
Transport to clinic	3	x	6/10	1.8
Stigma from in-laws	3	x	4/10	1.2
Total PGI score				3.0

The scores from each area were then summed up to generate a PGI score for each participant. The resulting score thus aimed to represent the extent to which reality falls short of patients' hopes and expectations in those areas of life in which they would most value an improvement [17].

The areas of importance listed by the participants were also clustered and summarized into themes through consensus by the authors.

The EQ-5D-3L results were summarized for each domain and an index score was obtained using weights from the validated EQ-5D-3L scores from Zimbabwe [30] since no validated weights for the EQ-5D-3L were available from a Kenyan population.

Summary scores from the PGI were correlated to the EQ-5D-3L index score using Pearson correlation.

### Ethics

Our study focused specifically on pregnant women living with HIV, and aimed to get an insight into issues affecting pregnant women living with HIV. Informed consent to participate in the study was sought from each of the participants by the recruiting team. The recruiting team consisted of the caregivers to the women and the research assistants. Entry and exit from the study was also clearly articulated to be voluntary to the participants. All methods were carried out following relevant guidelines and ethical regulations, and the study was approved by the Moi University Institutional Research and Ethics Committee (IREC 1292).

Trial Registration: ISRCTN98818734; registered on 9th December 2014.

## Results

The study sample consisted of 100 participants whose mean age was 29 years. The majority (62%) of the participants had disclosed their HIV status to their partners and could speak both Kiswahili and English Languages (60%). Their social demographics and clinical status are summarized in Table 2.

**Table 2:** Social demographics characteristics of the study participants

M	*N*	Frequency (%)
*HIV disclosure status*	100	
No		19 (19)
Yes		62 (62)
N/A (newly diagnosed with HIV)		19 (19%)
*Education*	100	
Primary		45 (45%)
Secondary		39 (39%)
Tertiary/vocational		12 (12%)
University		4 (4%)
*Languages*	100	
Both Kiswahili and English		60 (60%)
English		6 (6%)
Kiswahili		31 (31%)
Missing		3 (3%)
*Marital status*	100	
Divorced/separated		6 (6%)
Married with partner		80 (80%)
Single		11 (11%)
Widow		3 (3%)
*Occupation*	100	
Homemaker		36 (36%)
Casual laborer		8 (8%)
Employed		12 (12%)
Self-employed		18 (18%)
Student		3 (3%)
Unemployed		23 (23%)
*Total salary Ksh (USD)/month*	100	
1000–5000 ($10–50)		9 (9%)
5000–10,000 ($50–100)		9 (9%)
10,000–20,000 ($100–200)		8 (8%)
> 20,000 (> $200)		5 (5%)
Not certain/not willing to disclose/missing		69 (69%)
Age (years)	*Mean*, 29.63, Range (18–41)	

From the PGI questionnaire the women mentioned a total of 267 areas of importance to them that had been affected by their HIV status. Most of the women (64%) reported two (29%) to three (36%) areas of their lives affected by their HIV status against the request to list five areas. The areas mentioned were further summarized into thematic areas (Table 3). The areas of importance listed by the women mainly centered on themes of economic wellbeing (84%), physical health (58%), emotional health (49%), and relationships (28%). From the index score generated for each participant (n = 100), the mean PGI score was 2.01(SD ± 1.11). This score was an indication of the overall effect of their HIV status on their lives on a scale of 0- 6, with 6 being no effect and 0 being severely affected.

**Table 3:** Summary of areas of importance of the study participants affected by their HIV status and their patient-generated index mean score

Areas of importance by themes	Count of respondents
Economic well being	84
Physical Health	58
Emotional health	49
Relationship	28
Disclosure	23
Hospital visits	8
No area affected	7
Adherence	6
Sexual and reproductive health	3
Breastfeeding	1

*Mean PGI score* = 2.01, SD = 1.11

*Median PGI score* = 2.00, IQR = 0.0,5.80

The EQ-5D-3L results are summarized in Table 4. The majority of the women reported having no problems in any of the 5 domains in the EQ-5D-3L.

**Table 4:** Summary of the EQ-5D-3L domains among the study participants and mean score of the EQ-5D-3L index scores

EQ-5D-3L dimension	*n* = 100, Frequency		
	1 (no problems)	2 (some problems)	3 (severe/extreme)
Mobility	92	8	0
Self-care	95	5	0
Activity	90	10	0
Pain	81	19	0
Anxiety	82	17	1

*Mean* EQ-5D-3L Index Score = 0.93, SD = 0.11

*Median* EQ-5D-3L index score = 1.0, IQR = 0.59, 1.00

Similarly, the mean index score for the EQ-5D-3L was 0.93 [SD ± 0.11; n = 100] indicating a largely healthy sample of participants, given a score of 1 being perfect health.

From both the PGI and the EQ-5D-3L, the study participants had less than perfect health. However, from the correlation coefficient (0.01), there was a weak correlation between the PGI and EQ-5D-3L scores.

## Discussion

The use of the PGI among pregnant women living with HIV helped to highlight the perspective of the women living with HIV by lifting up areas of importance in their lives related to the infection. The majority of the women reported fewer than the 5 areas they were requested to list in the PGI, perhaps as a reflection of their overall health status, as also affirmed by the results of the EQ-5D-3L. While their overall health was relatively good, areas of interest revealed by the PGI are not routinely captured by the more commonly used EQ-5D-3L. These areas included: economic wellbeing (84%), physical health (58%), emotional health (49%), and relationship concerns (28%). These areas highlighted resonate with the broader definition of health by WHO that describes health as “a state of complete physical, mental, and social well-being and not merely the absence of disease or infirmity”. Also among people living with HIV, there has been a call to add a fourth 90 on HRQoL beyond the UNAIDS 90-90-90 goals, under two domains- comorbidities and self-perceived QoL [6].

Financial challenges are prominently highlighted by our study participants. A study in Ethiopia also found poor HRQoL among HIV-positive pregnant women with a low wealth index [31]. In our study, most of the participants did not respond to questions on household income. Among those who responded, 84% included economic wellbeing as an area of concern affected by their HIV status. This highlights the need for financial protection for pregnant women and other vulnerable groups when accessing health services. This further aligns to goal 3 of the United Nations sustainable development goals that aims to: “Ensure healthy lives and promote well-being for all at all ages”. Among the targets to achieve this goal is achieving universal health coverage, including financial risk protection [32].

Physical and emotional health was also listed by the women as an area of importance to them affected by their HIV status. This could perhaps be related to stigma and discrimination that is thought to also affect HRQoL among people living with HIV [33] Allowing the women to list what they perceived to be areas of importance to them, gave them a broader choice beyond the options provided in the EQ-5D-3L dimensions. In the EQ-5D-3L, 82% of the women reported having no anxiety, yet with the PGI, 49% reported emotional health and 28% reported relationships as areas of importance. These areas of importance highlighted by the women in the PGI may not find matching or comparable domains in the EQ-5D-3L.

These results from the use of the PGI among pregnant women living with HIV highlight the challenges of using a single tool in assessing HRQoL. In particular, while most women indicated they were of good health from the EQ-5D-3L, most indicated other areas of importance to them affected by their HIV status that were captured by the PGI. This is important to researchers studying the impact of interventions among patients. While standardized tools offer the advantage of comparing impacts across different interventions, addressing areas of importance to patients is critical in having a wholesome impact of interventions.

The areas listed by the study participants point to concerns often not well explored when assessing the impact of disease on individuals. While most attention is paid to the function of the individuals and interventions that restore this function, other areas that affect the wellbeing of the individuals are also important and need to be highlighted. This study serves to underscore this assertion. The use of the PGI in this study among pregnant women living with HIV served to highlight, from the patient perspective, areas of importance to the women that are negatively affected by HIV. This is critical in designing interventions considering the challenges that exist in offering care to this sub-population of patients. Patient perspectives are key in patient-centered health systems and interventions to provide health services should be responsive to the patient's need, beyond the therapeutic effects of the interventions. This study also further advances the use of the PGI tool in exploring areas of importance affected by a health condition among populations or diagnostic groups in a similar context,

### Study limitations

Our sudy has some limitations. Comprehension of the PGI tool by the respondents was difficult. For a tool to provide a valid and reliable measure, it is expected to be simple, easy to complete, and score [9]. To overcome this difficuclty, the tool was administered by the trained RAs to all the participants. This could potentially have introduced interviewer bias. To minimize this, the RAs were trained on administering the tools and piloting of the tools was done before administering them to the participants.

Compared to the EQ-5D-3L, the PGI presented a higher degree of difficulty in comprehension and filling out among the respondents. The abstract concept of spending points on areas of importance may not have been readily interpreted by respondents. This calls for adaptation of the tool to different social settings, especially those with low literacy levels. In addition, the tool would require validation for use in the Kenyan context. This would ensure all populations can use the tool and their views are captured as well to highlight the areas of importance in their lives affected by illness. Our study sample was limited to pregnant women living with HIV thus limiting the generalization of the results to this subpopulation. More assessments are welcome to other subpopulations of people living with HIV to understand their perspectives.

The EQ-5D-3L index score was obtained using weights from Zimbabwe since no validated weights from a Kenyan population were available at the time of the study. Despite acceptance and wide use of the EQ-5D-3L, the lack of validation of the tool for this population further demonstrates the limitations of generic tools in measuring HRQoL. Generic tools may also have limitations when used in certain cultural contexts where the tools have not been validated and some of the metrics assessed do not readily lend themselves to measurement. Simulation models for patient states may be an alternative approach to estimating quality-adjusted life years to be used in economic evaluation studies [34].

## Conclusion

The PGI may present a useful tool to assess areas of importance to pregnant women living with HIV. The PGI could potentially serve well to complement generic tools used to measure HRQoL in highlighting areas of importance to pregnant women living with HIV. This would make an important contribution to the 4th 90 goal of mental well-being and high quality of life among people living with HIV. Efforts should be made to simplify the use of the tools and make them more readily usable across different cultural and educational backgrounds. This is important to help patients voice their needs to the providers and policymakers, as patient-centered care and interventions are developed, to respond better to patient needs. Similar studies in other sub-populations would also be useful in understanding important areas of their lives affected by illness.

## Data Availability

The datasets used and/or analyzed during the current study are available from the corresponding author on request.

## Abbreviations

ART: Anti-retroviral therapy
EQ VAS: EuroQol- Visual Analogue scale
EQ-5D-3L: Euroqol-5 dimension-3level
HIV: Human immunodeficiency virus
HRQoL: Health-related Quality of Life
IQR: Interquartile range
IREC: Institutional Research and Ethics Committee
Ksh: Kenya Shillings
PGI: Patient Generated Index
QoL: Quality of Life
RAs: Research Assistants
SD: Standard deviation
UNAIDS: The Joint United Nations Programme on HIV/AIDS
USD: United States Dollar
WHO: World Health Organization
